# Kidney cancer characteristics and genotype-phenotype-correlations in Birt-Hogg-Dubé syndrome

**DOI:** 10.1371/journal.pone.0209504

**Published:** 2018-12-26

**Authors:** Elke C. Sattler, Marlene Reithmair, Ortrud K. Steinlein

**Affiliations:** 1 Department of Dermatology and Allergology, University Hospital, LMU Munich, Munich, Germany; 2 Institute of Human Genetics, University Hospital, LMU Munich, Munich, Germany; Agency for Science Technology and Research, SINGAPORE

## Abstract

Birt-Hogg-Dubé syndrome (BHDS) is a genetic tumor syndrome characterized by lung cysts, pneumothorax, fibrofolliculomas and renal cell cancer. The diagnosis of BHDS is usually considered if kidney cancer occurs before age 50 years, is multifocal and/or bilateral or of the oncocytoma/hybrid oncocytoma-chromophobe type. Using a sample of 50 BHDS families with a total of 178 patients we analyzed how many kidney cancer patients fulfilled one or more of these criteria. Furthermore, we addressed the question if genotype-phenotype-correlations exist that can be used for risk stratification. Renal cell cancer occurred in 34/178 (19.1%) patients, and the reported male bias was not observed. Furthermore, most kidney malignancies occurred after the age of 50 years. Thus, the majority of tumors did not show the typical hallmarks of BHDS. A below-average tumor frequency (17.2%) was observed for the known mutational hotspot c.1285delC/dupC that was the cause of BHDS in 24% of families. Unexpected was the high tumor frequency (66.7%) associated with mutation c.887C>G within a single family, a finding that merits further exploration.

## Introduction

Birt-Hogg-Dubé syndrome (BHDS, [MIM: 135150]) is an autosomal dominant tumor syndrome caused by mutations in the folliculin-encoding *FLCN* gene [1–4]. The disorder is characterized by symptoms affecting mainly skin, lung and kidneys that demonstrate high inter- and intrafamilial variability [5]. About 85% of the patients are found to have lung bullae that cause pneumothorax in about 25–30% of them [6]. Common skin symptoms in adult patients are benign hair follicle tumours named fibrofolliculomas that mainly develop on the face and neck, often steadily increasing in number and size during life time. A serious complication is renal cell cancer (RCC) that, in contrast to sporadic RCC, shows a strong trend towards multifocal and or bilateral manifestation. Histologically different types of kidney tumors are found, including clear cell, hybrid oncocytoma/chromophobe or papillary RCC. The reported life time risks for RCC in BHDS patients are between 14–35% [7–10]. Compared to the frequency of RCCs in the general population these risks are significantly increased. However they are lower than, for example, the 15–74% colon cancer risk in HNPCC (hereditary non polyposis colon cancer) or the 85% thyroid cancer risk in MEN2a (multiple endocrine neoplasia type 2a) [11]. The reasons behind the moderate RCC penetrance in BHDS are so far unknown. It is also mostly unknown which genetic or environmental factors modulate the tumor risk that leaves the majority of BHDS patients unaffected but cause multifocal and/or bilateral RCC in others. Previous studies have already presented data about RCC risks and *FLCN* mutations but did not focus specifically on genotype-phenotype-correlations [12]. In the present study we therefore analyzed possible genotype-phenotype-correlations that might affect RCC penetrance in BHDS patients. We indeed found correlations that are suggestive of increased tumor risks for certain BHDS patients. Furthermore, we demonstrate that only a minority of BHDS patients develops one or more of the clinical features that are regarded as typical for this inherited tumor syndrome.

## Materials and methods

### Patients

All research was conducted according to the declaration of Helsinki principles. The study has been approved of by the ethical committee/institutional review board (IRB) of the Medical Faculty, University Hospital Munich, under the project-number: 489/16UE. Informed consent was obtained from all individuals participating in DNA testing. Detailed clinical data were obtained from 178 BHDS patients (male 86, female 92) belonging to 50 unrelated families. A subset of 83 patients was available for germline *FLCN* testing, and additional family members were only included if they had at least two major BHD symptoms or were obligate carriers. 42 families were of German origin, one each from Turkey, Switzerland and Greece, two from Great Britain and three of Eastern European origin with German roots. Only family members >18 years of age were included because children are usually not tested for BHDS and rarely develop the typical symptoms.

### Genetic testing

The complete *FLCN* gene coding region including adjacent intronic sequences was screened for mutations by PCR and subsequent Sanger sequencing following standard protocols (for primer details and PCR protocols see S1 Table). For each PCR 50–100 ng DNA were amplified using the HotStarTaq DNA Polymerase and Invitrogen^TM^Taq DNA Polymerase recombinant (Qiagen, Hilden, Germany; Thermo Fisher Scientific, Dreieich, Germany). Purification of the amplification products was performed with the Qiagen PCR purification kit (Qiagen, Hilden, Germany), and PCR products were sequenced using the 3500 Genetic Analyser (Thermo Fisher Scientific, Dreieich, Germany). MLPA (multiplex ligation-dependent probe amplification) was performed using the SALSA MLPA P256 *FLCN* probemix (MRC Holland, Amsterdam, The Netherlands) according to the manufacturer’s protocol on the ABI 3100 Avant (Applied Biosystems, Darmstadt, Germany) and analyzed by Coffalyser.Net software (MRC Holland, Amsterdam, The Netherlands). Statistical analyses were performed using the two-tailed Mann-Whitney U Test and the Chi-square Test.

## Results

RCCs were diagnosed in 20/50 BHDS pedigrees (40.0%). In 7/20 (35.0%) of these families more than one family member was affected. RCCs were diagnosed in 34 patients, and were bilateral (synchron or metachron) in at least six of them. No sex differences were observed regarding the number of BHDS patients that developed RCCs (females 17/34 (50.0%) versus males 17/34 (50.0%), or with respect to the age at initial diagnosis (female: median 54.5 years, range 42 years; male: median 57.0 years, range 50 years; Z-Score -0.58199, U-value 97.5, p .56). The average age of onset is well below that of sporadic RCC (61.8 years) [12–16–17](Table 1). Previously BHDS families have been reported that showed a RCC-only phenotype without further typical symptoms [14]. None of our 50 families showed this phenomenon; they all had at least one family member with fibrofolliculomas or pneumothorax.

**Table 1:** Patients with RCCs: Histological type and age of diagnosis.

Patient ID^1^	Histology	Age (y)	Sex
BHD15-II1	NA	72	M
BHD15-III1	NA	30	M
BHD15-IV1	hybrid oncocytoma/chromophobe RCC, unilateral	45	M
BDH17-II1	NA	65	M
BHD20-II1	chromophobe RCC, multiple unilateral	56	F
BHD20-II4	chromophobe RCC, single	56	F
BHD20-III5	hybrid oncocytoma/chromophobe RCC, unilateral	37	F
BHD21-III3	clear cell RCC bifocal unilateral,	49	F
BHD23-III1	chromophobe RCC, bilateral	46	F
BHD23-IV1	NA, bilateral	NN	M
BHD24-III3	NA	70	F
BDH26-IV2	clear cell RCC, unilateral	43	M
BDH26-IV3	NN	43	F
BHD28-III3	NA	NN	M
BHD30-III3	chromophobe RCC, bilateral multiple	50	M
BHD31-III4	chromophobe, unilateral	55	F
BHD33-III5	clear cell RCC, unilateral	35	M
BHD33-IV5	clear cell RCC, bilateral, metachron	69+79	M
BHD35-II2	NA	NN	F
BHD36-IV8	NA	NN	M
BHD38-II2	NA	59	F
BHD38-III2	NA	72	F
BHD38-IV2	chromophobe RCC, unilateral	56	F
BHD40-II3	clear cell RCC, bilateral	80	M
BHD45-II7	NA	46	F
BHD45-II9	clear cell RCC, unilateral	79	F
BHD45-III3	bilateral, metastatic	66	M
BHD45-II7	hybrid oncocytoma/chromophobe, unilateral	64	M
BHD45-II7	NN	57	M
BHD45-II7	papillary, unilateral	54	F
BHD46-III1	clear cell, bilateral hybrid papillary/chromophobe, unilateral	45 57	F
BHD47-IV2	hybrid oncocytoma/chromophobe, unilateral	39	F
BHD48-II4	NN	57	M
BHD50-IV5	clear cell, unilateral	50	M

^1^ Given are the family ID followed by the pedigree number. NA, not available because diagnosis was made >10 years ago which is the statutory period of retention for medical documents. NN, patient did not agree to make medical reports available.

**Fig 1:**
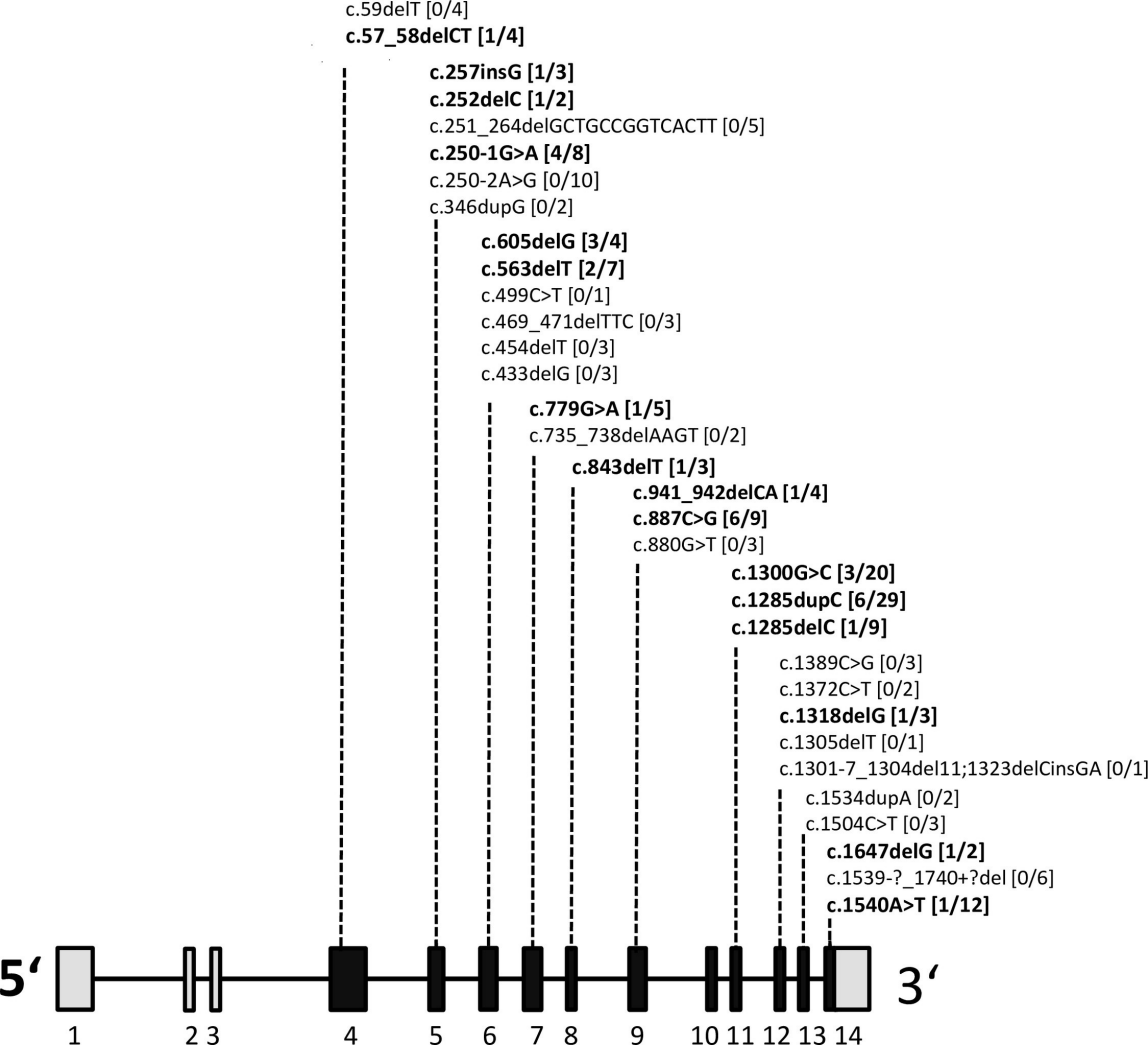
*FLCN* mutations and kidney cancer. Schematic presentation of coding (black bars) and non-coding (grey bars) *FLCN* exons. The positions of germline mutations detected in the BHDS sample are given. Mutations associated with RCCs are given in bold. Square brackets contain the numbers of patients that developed RCC, followed by the total number of BHDS patients. Exon numbering is shown at the bottom.

As demonstrated in Fig 1, a total of 33 different *FLCN* germline mutations were found. The heterozygous mutations were either frame shifting or truncating and are predicted to result in nonsense mediated decay or in a nonfunctional protein. The *FLCN* mutations were distributed over all but one (exon 10) of the 11 coding exons. In one family that was clinically diagnosed with typical BHDS no *FLCN* mutation was detectable. These results are in accordance with the reported mutation detection rates of 81–96% [12–13,18].

The tumors were associated with 16 distinct mutations in 9 different exons. At a first glance RCCs seemed to be disproportionately often associated with mutations in exon 11. However, this exon contains a known mutational hotspot (NM_144997.6: c.1285C) that in our sample was the cause of BHDS in 12/50 pedigrees [13,19]. Taking the total number of patients per exon into consideration the RCC frequency for exon 11 was actually lower than that of most other exons (17.2%, compared to 20.0% for exon 5 or 23.8% for exon 6).

We were not able to confirm previous reports that described an up to 2.5 fold increase of RCCs in male compared to female BHDS patients [12–15]. There were no differences regarding the age at which an RCC was diagnosed between mutations located in the 5’- or 3’-part of the *FLCN* gene (51.9±13.2 vs 55.3±10.7 years, Z-Score -0.75056, U-value 58.5, p 0.45326). However, the position of the mutation within the gene might be a factor influencing cancer risk. This would be unlikely in disorders that are caused by haploinsufficiency, because all mutations would result in the same loss of function. However, it could be an important factor if the mutated *FLCN* mRNA is translated and the truncated protein acts in a dominant negative manner. Proteins truncated within the 5’ part or 3’part differ with respect to their total length and remaining functional domains. So far it is not sure which of the two mechanisms applies to FLCN, which renders it reasonable to investigate possible phenotype differences between 5`and 3`mutations. The frequency of RCCs with respect to the total number of patients per exon showed a higher RCC rate for patients with a mutation located in the 5’ part (exon 4–8, 14/69 patients, 20.3%, total subgroup: 56.1±16.2 years) compared to those with a mutation in the 3’ part (exon 10–14, 13/93 patients; 14.0%, total subgroup: 57.4±16.3 years) of *FLCN* (p-value 0.30, odds ratio 1.56197, 95% CI 0.63–3.92). These differences are not significant; larger samples are needed to decide if genotype-phenotype correlations exist with respect to the intragenic position of mutations.

## Discussion

Exon 9 was not included in the genotype-phenotype analysis because it would have distorted the results. Mutation *FLCN* c.887C>G in exon 9 stands out because it was found in a family (BHD45) in which six out of nine (66.7%) patients developed RCCs. This unusually high rate of RCC might be due to unknown genetic or environmental risk factors within this specific family, but a true genotype-phenotype effect can also not be excluded yet. It therefore seems prudent to look out for additional families carrying this specific *FLCN* mutation.

It has been suggested that BHDS (as well as other genetic tumor syndromes) should be considered if an RCC occurs before age 50 years [17]. However in our sample 16/25 (64.0%) patients were diagnosed with RCC at age 50 years or older (only patients included for which this information was available). The criterion ‘age’ alone would have therefore been insufficient as an identifier for BHDS. Adding the criterion ‘bilateral or multifocal RCC’ would have raised a red flag only for six patients from the 50+ age group (37.5%). Bilateral or multifocal tumor presentation also was not a common finding in the 18–49 years age group (37.5%). The diagnostic criteria proposed by the European BHDS Consortium also include RCC histology, with a chromophobe or hybrid chromophobe-oncocytoma cancer subtype being considered suspicious of BHDS [7]. Previous reports found the chromophobe tumor type in about 34% of BHDS-RCCs, which is a good match for the 38.1% frequency this tumor type had in our sample [12,15,20–21]. However, the hybrid chromophobe-oncocytoma cancer subtype was reported to have a 50% frequency in BHDS-RCCs but in our sample only 19.1% of RCCs for which histological information was available showed this subtype [22]. The clear cell RCC (47.6%) was the most common histological subtype in our sample. These findings deviate from earlier reports in which clear cell RCCs only accounted for about 9% of all renal tumors in BHDS patients [12,15,20, 22–23]. These discrepancies could very well point towards a methological problem. Chromophobe tumors are prone to be misdiagnosed as clear cell RCCs if not analysed by specialised uropathologists. It would therefore have been preferable to have all tumor samples analysed by the same team of specialists. Unfortunately this was not managable because many tumor samples were no longer available and we often had to rely on the written reports. It is therefore questionable whether our numbers reflect the true frequencies of the different RCC subtypes. On the other hand ascertainment bias regarding the published frequencies cannot be excluded. Unusual RCC subtypes such as hybrid chromophobe-oncocytomata are more likely to result in a referral to a BHDS centre, resulting in an overestimation of this RCC subtype. This bias probably also exists in our sample, however, the majority of our patients was referred to our BHDS centre because of their dermatological or pneumological health problems and are therefore unlikely to be profoundly affected by this bias. Another contributing factor could be that clear cell-like cytoplasm and prominent perinuclear halo are frequent features in different types of RCC in BHDS. These features can be misinterpreted in routine histopathological assessment, resulting in overrepresentation of clear cell RCC diagnosis [23].

BHDS patients have substantial health risks but regular radiological and dermatological check-ups can considerably lower their morbidity and mortality rates. It is therefore crucial that the diagnosis of BHDS is made as early as possible and that the index patient is encouraged to inform the family members about the possibility of genetic counselling and predictive testing. In our sample the criteria age of onset >50 years, chromophobe/hybrid chromophobe-oncocytoma tumor histology or multifocal/bilateral tumor localisation would have raised suspicion of BHDS only in a minority of patients. This emphasizes that an interdisciplinary approach is needed to identify patients with this rare tumor syndrome. The most effective approach includes a careful dermatological inspection, a medical history focusing on renal and pulmonary problems and a thorough pedigree analysis covering at least three generations. This approach greatly increases the chances to recognize patterns of symptoms in multisystem disorders such as BHDS. Genetic testing can then be used to confirm the diagnosis in the index patient by detecting the underlying *FLCN* mutation. Once known this mutation offers the chance to identify additional family members at risk. The intragenic location of the *FLCN* mutation does not yet influence tumor screening strategies or treatment planning. The potential use of genotype-phenotype correlations for the improvement of patient care needs to be analyzed in independent BHDS samples.

## Supporting information

S1 Table FLCN–PCR: primer sequences and conditions. (DOCX)
