# Supplementary material for: Kidney cancer characteristics and genotype-phenotype-correlations in Birt-Hogg-Dubé syndrome
Source: PLoS One. 2018 Dec 26;13(12):e0209504. doi: 10.1371/journal.pone.0209504 (PMC6306193; doi:10.1371/journal.pone.0209504)
Supplement: S1 Table — (DOCX) [file pone.0209504.s001.docx]

**Table S1**

**FLCN–PCR: primer sequences and conditions**

Exon Primer annealing temp.(°C)

4 ggggaggtttcatggagtc

tgtccatcccacacctactg 59,2

5 ccctgcttcccaactaacag

tgcctccctgtgcaatgctg 59,2

6 cactaagcgaggaaagggctg

tctccaggcctcaacctcag 66,1

7 ggagttggctgtgaacgag

tgactgctctatcctaacag 55,4

8 gccctgctggtgttctttta

ctgccaggagagcagacag 59,2

9 gaacagctgacggtctttcc

atgactggctctcctcctga 57,7

10 ctagtcacgctgaaagcactg

gtgctgggcagtcggtgcac 63,9

11 gggtagtagagcatggatg

gaacctcagcgcagggcatg 59,2

12-13 ccccactgacctgggatgag

gcaaaggggcctcacccacac 66,1

14 gctggtgccaaagccgtgtc

ccttccagcagttgagaaact 63,9
